# Ozone Sensitivity and Catalase Activity in Pigmented and Non-Pigmented Strains of *Serratia Marcescens*

**DOI:** 10.2174/1874285801711010012

**Published:** 2017-03-31

**Authors:** José de Ondarza

**Affiliations:** Department of Biological Sciences, Plattsburgh State University of New York, NY, USA

**Keywords:** Catalase, oxyR, ozone, pigment, peroxide, prodigiosin, Serratia

## Abstract

**Background:**

Ozone exposure rapidly leads to bacterial death, making ozone an effective disinfectant in food industry and health care arena. However, microbial defenses may moderate this effect and play a role in the effective use of oxidizing agents for disinfection. *Serratia marcescens* is an opportunistic pathogen, expressing genes differentially during infection of a human host. A better understanding of regulatory systems that control expression of *Serratia*’s virulence genes and defenses is therefore valuable.

**Objective:**

Here, we investigated the role of pigmentation and catalase in *Serratia marcescens* on survival to ozone exposure.

**Method:**

Pigmented and non-pigmented strains of *Serratia marcescens* were cultured to exponential or stationary phase and exposed to 5 ppm of gaseous ozone for 2.5 – 10 minutes. Survival was calculated via plate counts. Catalase activity was measured photometrically and tolerance to hydrogen peroxide was assayed by disk-diffusion.

**Results:**

Exposure of *S. marcescens* to 5 ppm gaseous ozone kills > 90% of cells within 10 minutes in a time and concentration-dependent manner. Although pigmented *Serratia* (grown at 28°C) survived ozonation better than unpigmented *Serratia* (grown at 35°C), non-pigmented mutant strains of *Serratia* had similar ozone survival rates, catalase activity and H_2_O_2_ tolerance as wild type strains. Rather, ozone survival and catalase activity were elevated in 6 hour cultures compared to 48 hour cultures.

**Conclusion:**

Our studies did not bear out a role for prodigiosin in ozone survival. Rather, induction of oxidative stress responses during exponential growth increased both catalase activity and ozone survival in both pigmented and unpigmented *S. marcescens*.

## INTRODUCTION

Ozone is a potent oxidizing agent that occurs naturally in the atmosphere at low (< 50 ppb) concentrations, but can rise to levels as high as 200 – 300 ppb in smog. At such levels, ozone becomes a significant health risk associated with respiratory irritation. At yet higher levels, its oxidizing potential can be life-threatening, but also serve as a useful disinfection agent with applications in food industry [1], water quality maintenance [2], and in the health care industry [3, 4]. The efficacy of ozone as a disinfecting agent has been reported to also vary significantly based on the concentration, time of exposure, presence of organic materials, and type of microbe exposed, with up to a 4-log reduction in pathogen counts on seeds reported at concentrations of 2000 ppm [5], although most treatment approaches use lower concentrations of 0.1 – 10 ppm with correspondingly lower reductions in microbial counts [6, 7]. A wide variety of pathogens respond differently to ozone disinfection, raising the question of whether sensitivity to ozone can be moderated by specific microbial defenses such as catalase, superoxide dismutase, or other antioxidant systems. Indeed, microbial responses such as elevation of SOD and catalase activity have been demonstrated in *Listeria* [8] and *E. coli* [9] in response to ozone exposure. Microbial responses to hydrogen peroxide exposure are better studied and reveal much of the underlying regulatory mechanisms, including induction of catalase, alkyl hydroperoxidase, and superoxide dismutase by oxidative stress via transactivator molecules such as oxyR [10, 11], rpoS [12] and soxRS [13]. Alternatively, microbial pigmentation offers another potential defense against environmental challenges, including predation [14, 15], competition (antibiotic effects), and potentially, oxygen radicals [16-18]. Such pigments are often produced by bacteria as secondary metabolites via a quorum-sensing mechanism, including prodigiosin and violacein [19-21]. Whereas the purple pigment violacein can function as an anti-oxidant [22], such a role has yet to be demonstrated for prodigiosin, a red pigment produced by *Serratia marcescens*. *Serratia*, a Gram-negative, ubiquitous microbe and opportunistic human pathogen, produces the red pigment prodigiosin in a temperature and quorum sensing-dependent manner, and possibly benefits from such pigments due to their antibiotic properties, although the exact function of these has not been completely elucidated [17]. The pigment (prodigiosin) is thought to be located in the cell wall of the bacteria and contributes to cell surface hydrophobicity [23, 24], in a location where it could also conceivably act as a first layer of defense against external oxidative challenges. Alternately, it has been proposed that prodigiosin may act as an energy-spilling role, reducing ATP synthesis, oxidative metabolism and thus, reactive oxygen species generation [25]. We therefore sought to determine if pigmentation also offers a survival advantage and a defense against exposure to ozone. We show here that ozone kills *Serratia marcescens* in a time-dependent manner and that growth phase of the cultures, but not pigmentation or oxyR, affect survival to ozone exposure.

## MATERIALS AND METHOD

### Bacterial Strains

*Serratia marcescens* (wild type) was purchased from Presque Isle Cultures (Erie PA). All other *Serratia marcescens* strains were kindly provided by Dr. Robert Shanks (University of Pittsburgh) (Table **1**).

### Culture

All strains were initially grown in Tryptic Soy Broth (Hardy Diagnostics, Santa Maria CA) prior to plating on MacConkey agar (Becton Dickinson, Franklin Lakes NJ) for ozone exposure. Wild-type *S. marcescens* was cultured for 48h at either 30°C (red-pigmented, SM-R) or 37°C, a temperature at which prodigiosin is not produced (SM-W). *S. marcescens* strains SM-01, SM-02, SM-04, SM-05, and SM-06 were cultured 6h (exponential phase) or 48h (stationary phase) at 30°C, yielding pigmentation in wild-type and oxyR mutants but not the pigA deletion mutants. Pigmentation level was assayed as the ratio of light absorbance at 535 nm/600 nm. 1 ml of each 48h cell culture was centrifuged at 13,000g to pellet cells, supernatant discarded, and pigment extracted in 1 ml acid-alcohol (95% isopropanol + 1% 2N HCl). A_600_ was measured for 500 µl of cell culture resuspended in 2 ml saline; A_535_ was measured for 500µl pigment extract resuspended in 2 ml dH_2_O.

### Ozone Exposure

Growing (6h) or stationary (48h) cultures of *S. marcescens* were diluted in TSB to an Absorbance of 0.100 at 600 nm, serial-diluted to 10^-4^ or 10^-5^ in 0.9% saline, and spread-plated onto 125 mm MacConkey agar plates. Agar plates were then exposed to 5 or 10 ppm ozone inside a 42L plexiglass chamber. Ozone was generated with an OZX-300 Ozone generator (Enaly Co, Shanghai, China) and continuously monitored with a Model 106-L Ozone Monitor (2B Technologies, Boulder CO). Concentrations were maintained to within 0.5 ppm. Exposure times ranged from 2.5 minutes to 20 minutes. Agar plates were subsequently incubated for 24 - 48h at room temperature, colonies counted, and survival calculated as a percentage of unexposed controls.

### Pre-Exposure of Serratia to H_2_O_2_

 Exponentially growing 5 ml TSB cultures of *S. marcescens* were subjected to oxidative stress by pre-exposure to low (200 µM) or high (20 mM) levels of H_2_O_2_ for 30 min prior to ozone exposure and catalase measurements.

### H_2_O_2_ Disk Diffusion Assay

 Sensitivity of *S. marcescens* to H_2_O_2_ was determined by disk-diffusion assay. Pour plates were prepared by mixing 100μl of a 0.500 Absorbance (600 nm) culture broth of each test strain with 2 ml of melted (45°C) Top Agar (50% Trypticase soy agar/50% Tryticase soy broth) and pouring it over bottom agar (10 ml TSA in 48 mm sterile Petri plates). Once solidified, sterile 6 mm blank paper disks were placed on the agar surface and 10 µl of 30% H_2_O_2_ (Sigma-Aldrich) was added to each disk. Plates were incubated for 24h at either 28°C or 37°C and zones of inhibition measured. Each assay was conducted at least 3 separate times on different days.

### Catalase Assay

 Catalase activity was assayed following the methods of Beers [26]. In brief, 1 ml of each SM broth culture was pelleted by centrifugation at 6,000 x g, washed and resuspended in 2 ml of 0.9% saline, and sonicated at 40% power twelve times (30s on/30s off) on ice. Cell lysates were centrifuged for 20 min at 12000 x g to pellet cell debris, and supernatants were collected. 20µl of each lysate, supernatant, and pellet (resuspended in 1 ml saline) were then added to 50 mM phosphate buffer (pH 7) containing 30 mM H_2_O_2_. Occasionally, 100 μl lysate samples were heated to either 50°C or 100°C for 20 minutes before assaying. Catalase activity was measured by following the change in absorbance at 240 nm in an Ocean Optics 4400 UV/Vis spectrometer and calculated as (ΔA240/min)/(43.6/Mol/min * sample volume * 0.001). Catalase activity was normalized to sample protein concentration when possible using Bradford protein assay.

### Statistical Analysis

 Pairwise comparisons were performed using Student’s t-test, while multiple comparisons were conducted using one-way ANOVA with GraphPad Prism software.

## RESULTS

### Survival to Ozone Exposure

**Fig. (1):**
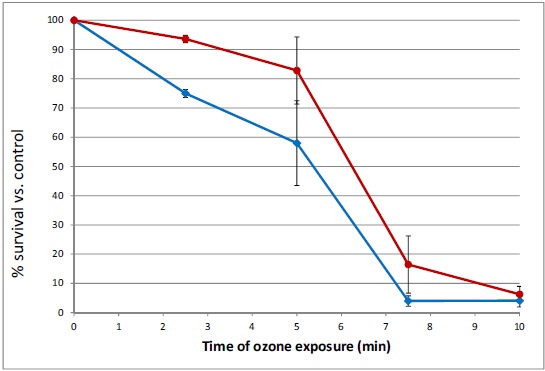
Survival of *Serratia marcescens* after exposure to 2.5 – 10 min of gaseous ozone (5 ppm). Pigmented SM-R (open squares) survived ozone exposure better than unpigmented SM-W (filled diamonds); * significantly different (P < 0.05).

**Fig. (2):**
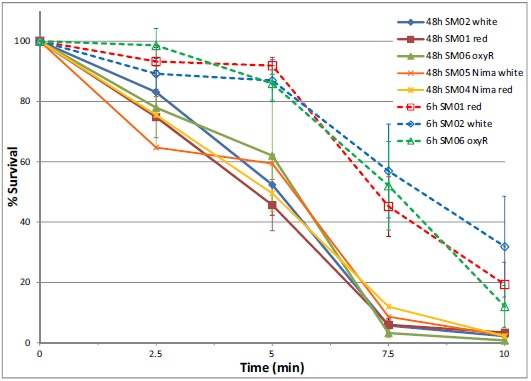
Survival of wild type and mutant *S. marcescens* strains following ozone (5 ppm) exposure. *S. marcescens* strains were incubated for either 6h or 48h prior to ozonation. No significant differences were observed among strains in stationary phase (48h) or among strains in exponential phase (6h), but survival was significantly greater (P < 0.05) for exponential-phase cultures compared to stationary-phase cultures.

Exposure of 48 h cultures of *Serratia marcescens* to ozone showed a time-dependent and concentration-dependent reduction in viable cell numbers for both pigmented and unpigmented strains. Initially, brief 30 second exposures to a range of ozone concentrations were tested to determine effective concentrations for short-term (5 – 30 minute) exposures. Based on these results (data not shown), concentrations of 5 and 10 ppm were selected. Low levels of ozone (5 ppm) significantly reduced bacterial numbers over a 10 minute time period, affecting non-pigmented *S. marcescens* (SM-W) more than pigmented strains (SM-R) (Fig. **1**). This effect was significant at 2.5 min exposure (P<0.05), but also pronounced at 5 and 7.5 min. Similarly, ozonation at 10 ppm reduced SM-01 cell numbers, but at a faster rate (10-fold reduction in 5 min). Pigmentation in 48h broth cultures of SM-R (cultured at 30°C) was robust, attaining an Absorbance ratio (A534/A600) of 0.472, whereas the same strain grown at 37°C exhibited no pigmentation (A535/A600 ratio = 0.005). To clarify whether pigmentation, or some other change brought about by lower incubation temperature, was responsible for the lower sensitivity to ozone, this experiment was repeated using two wild-type *S. marcescens* strains (SM-01 and SM-04 Nima) and their pigA deletion mutants (SM-02 and SM-05, respectively), with incubation conditions maintained at 30°C. Both mutant strains exhibited similar survival following 5 ppm ozone exposure as their pigmented counterparts (Fig. **2**), strongly suggesting that pigmentation may not be responsible for the observed difference in sensitivity to ozone.

### Sensitivity to Hydrogen Peroxide Exposure

**Fig. (3):**
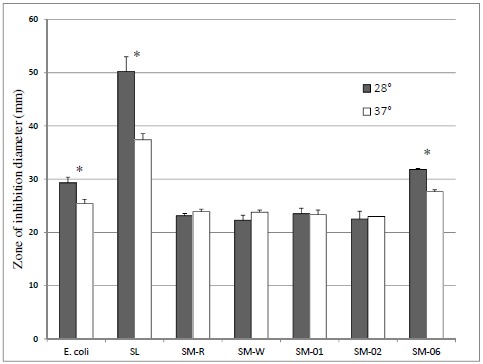
**Effect of assay temperature on H_2_O_2_ sensitivity** (Zone of inhibition diameter) for *E. coli*, *Streptococcus lactis* (SL), and *S. marcescens* strains SM-01, SM-02, SM-06 (all cultured at 30°C) and SM wild type cultured at either 28°C (SM-R) or 37°C (SM-W) before exposure to 10µl H_2_O_2_. Agar plates + H_2_O_2_-impregnated disks were incubated for 24h at 28°C or 37°C. Incubation at pigment-producing temperature (28°C) slightly increased SM resistance to H_2_O_2_ exposure, whereas *E. coli*, catalase-lacking *Streptococcus lactis* (SL), and the oxyR mutant of *Serratia* (SM-06) are more sensitive (P < 0.05) at this temperature. * significantly different (28°C vs 37°C).

 To investigate whether expression of anti-oxidant enzymes such as catalase mediate this protection against ozone exposure, SM-R and SM-W were suspended in top agar and pour-plated onto a bottom agar base prior to incubation in the presence of 10µl of 30% H_2_O_2_ in a disk-diffusion assay. Although SM-R had slightly smaller inhibition zones when assayed at pigment-producing temperature (28°C) than SM-W assayed at 37°C (Fig. **3**), this difference was not significant. As well, SM-W also showed slightly higher (P > 0.05) resistance to H_2_O_2_ at 28°C, and greater resistance to H_2_O_2_ than SM-R at each assay temperature. This suggests that SM-W has better peroxide defenses (and potentially) catalase activity. A similar pattern was observed when comparing the SM-04 (Nima) strain to its pigA deletion counterpart (SM-05), with pigmented SM-04 having a similar, though slightly smaller, inhibition zone than unpigmented SM-05 at 28°C, or SM-04 assayed at 37°C (unpigmented). In comparison, both *E. coli* (known to have multiple catalases) and *Streptococcus lactis* (lacking catalase) have significantly smaller inhibition zones at 37°C (Fig. **3**), as does an oxyR mutant of *Serratia* (SM-06).

### Catalase Activity in Serratia Strains

**Fig. (4):**
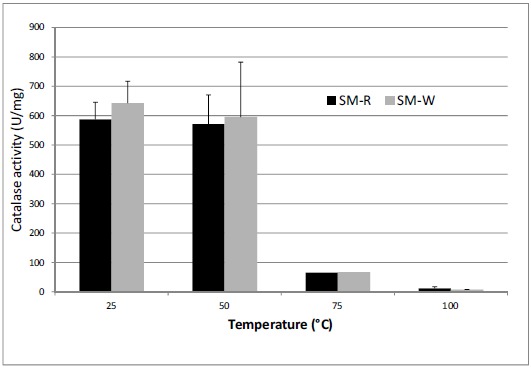
Catalase activity (U/mg) of pigmented (red) and unpigmented (white) *Serratia marcescens* cell lysates, normalized to protein concentrations. Heating of lysates to 50°C did not affect catalase activity, but activity was significantly lower in lysates heated to 75°C or 100°C (P < 0.05). For temperature stability assays, only cell lysates were used.

 To assess catalase activity directly, H_2_O_2_ degradation was measured spectrophotometrically. Consistent with the disk diffusion data, SM-W (grown at 37°C) exhibited slightly higher catalase activity than SM-R (grown at 28°C) (Table **2**). Catalase activity was detected in the cell lysate following sonication, in the soluble fraction (supernatant) of the lysate following centrifugation, in heated (50°C) lysate and in intact cells, but not in the pelleted cell debris or in boiled supernatant (Fig. **4**). Heating of cell lysates to 50°C reduced catalase activity by 3% - 15% in all strains examined, whereas heating to 75°C reduced activity by 84% - 90% and boiling reduced activity essentially to background levels. Catalase activity was also shown to be slightly greater in the non-pigmented SM-02 pigA mutant than in the wild type strain SM-01.

### Sensitivity to Ozone and H_2_O_2_, and Catalase Activity, of oxyR Mutant *S. Marcescens*

 To further investigate the role of oxidative stress defenses in ozone sensitivity and catalase activity, an oxyR mutant strain [11] was assayed. OxyR serves as a sensor of oxidative stress in many bacteria and regulates expression of catalase [13]. 48h cultures of SM-06 (OxyR mutant) had similar ozone survival (Fig. **2**) and catalase activity (Table **2**) as the wild-type (SM-01) strain even while maintaining their greater sensitivity to H_2_O_2_ exposure in a disk-diffusion assay as previously established [11]. Interestingly, the oxyR mutant exhibited a lower sensitivity to H_2_O_2_ at 37°C, much like *E. coli* and *S. lactis* (Fig. **3**) and unlike *Serratia* strains SM-01 and SM-02.

### Effect of H_2_O_2_ Pre-Exposure on Ozone Sensitivity and Catalase Activity

Since the putative function of oxyR is to up-regulate microbial defenses against oxidative stress, we then repeated the ozone and catalase assays in *Serratia* strains which were cultured for 6h (exponential phase) and then pre-exposed to oxidative stress (200 μM or 20 mM H_2_O_2_). Catalase activity in 6h cultures was significantly greater than that of 48h cultures in SM-01 (pigmented) and SM-02 (non-pigmented), but not in SM-06 (oxyR mutant) strains of *S. marcescens* (Table **2**). However, no significant differences were observed between catalase activity of H_2_O_2_-pre-exposed cultures and controls (P > 0.48). We also examined the effect of H_2_O_2_ pre-exposure in 6h cultures on *S. marcescens* sensitivity to ozone exposure. All strains of *Serratia* tested after 6h of incubation (SM-01, 02 and 06) exhibited a significantly greater survival to ozonation at 5 ppm than any stationary phase (48h culture) *Serratia* strains (Fig. **2**). Pre-exposure to H_2_O_2_ had a slight but not significant effect on ozone survival, with slightly better 5 min survival rates for 6h cultures pre-exposed to 50mM H_2_O_2_.

## DISCUSSION

### Effect of Ozone Exposure on S. Marcescens

Ozone is a potent and highly reactive molecule which can rapidly induce cellular damage in bacteria as well as humans. Its potent antibacterial action has led to its resurgence as a disinfectant in industrial, medical and food industry settings [2]. Because it is naturally occurring, we thought it plausible that bacterial defenses against oxidizing agents will protect them from ozone disinfection. Here, we initially showed that continuous exposure to 5 – 10 ppm of gaseous ozone kills *S. marcescens* in a time and concentration-dependent manner, and that these reductions are in line with reported ozone killing efficiencies in fruits and vegetables [6, 27, 28] where microbes attached to food surfaces were effectively killed by 0.1 – 9.2 ppm of ozone. Although up to 4-log reductions were observed with dry seed surfaces [5], the above-mentioned studies reported more typical log_10_ reductions of 0.22 – 2.89 for pathogens on moist food surfaces, depending on bacterial strain and exposure time. In the present study, *Serratia* cells were attached to a moist (agar) surface, and exposure times were similar or shorter (2.5 to 10 min vs. 5 min to 6 h) than in previous studies, but in general we attained a similar 1 – 2 log reduction in 10 min at 5 ppm (Ct value = 50 ppm.min) as with *Salmonella* and *E. coli* on bell peppers treated for 30 min at 1 ppm [27], *E. coli* on carrots and lettuce treated for 15 min at 5 ppm [28], and coliforms on papaya treated for 20 min at 9.2 ppm [6]. Ozone is thought to disrupt bacterial cell envelopes, leading to cell death by cytoplasmic leakage and cell lysis [1, 27].

### Effect of Pigmentation on Survival to Ozone Exposure

Unlike most other coliforms, *Serratia marcescens* is capable of producing the red pigment prodigiosin. Expression of prodigiosin is under complex regulatory control; its genes are part of the *pig* operon (*pig* for pigment) which allows pigment production to be regulated in a temperature and quorum-sensing-dependent manner [17, 20]. Our observation of greater survival of pigmented *Serratia* cells to ozone exposure than cells of the same strain that are unpigmented (when cultured at higher temperatures) could therefore be explained either by the presence of pigment or by differential expression of other protective factors at the lower temperature. Cultures of wild-type SM grown at 30°C for 48h were intensely pigmented; this pigmentation is presumably located in the bacterial wall [17] and thus present in single cells of pigmented, but not unpigmented, cultures. Such cells consistently survived ozone exposure better than unpigmented cells of the same strain cultured at 37°C (Fig. **1**). However, neither of the pigA-mutant strains of *Serratia* differed from their isogenic pigmented strains in term of ozone survival, and it is therefore likely that a temperature-induced change in protective genes could be responsible. The transition from environmental temperatures to that of an animal host is known to induce significant alterations in gene expression in many pathogens, including dimorphic fungi and environmental bacteria such as *Listeria* [29], *Pseudomonas aeruginosa* [30], and *Serratia,* where porin production [31], pigmentation, and possibly catalase expression [32] are changed.

### Possible Role of Catalase in Ozone Resistance

 Catalase expression is of particular interest, as catalase activity is affected by ozone exposure [8, 33], and we therefore examined more closely the role of catalase in *Serratia*. Genes for monofunctional (HPII; katE, 478 aa) and bifunctional (HPI; *katG*, 731 aa) catalase have been sequenced in *Serratia* [34, 35]. Presumably, then, both constitutive and inducible forms of catalase operate in *Serratia*. Direct measurement of catalase activity show similar levels in pigmented (SM-R, SM-01) and non-pigmented (SM-W and SM-02) strains cultured for 2 or more days (Table **2**). This catalase activity is likely due to the monofunctional catalase (HPII), which, at least in *E. coli*, is increasingly expressed as cells transition into stationary phase [36]. This is consistent with detection of monofunctional catalase activity in *S. marcescens* [37, 38]. The catalase described by Zeng [38] is also relatively stable up to 70°C, consistent with our data (Fig. **2**). The HPI form of catalase, in contrast, is a bifunctional catalase that is encoded by katG and is under control of the OxyR regulon in *E. coli* [13, 39, 40], an operon which also functions in *Serratia marcescens* [11]. Although this enzyme was not directly detected in *Serratia* in either mid-log or stationary phase cells [37], exposure of *Serratia* cells to 50 mM H_2_O_2_ did indeed induce katG expression dramatically in the recently described high oxidative stress-tolerant isolate *Serratia* sp. LCN16, a response which was absent in oxyR mutants [41].

### Response of S. Marcescens to Oxidative Stress (H_2_O_2_ Exposure)

Here, we were unable to observe a significant increase in catalase activity following low (200 μM) or high (20 mM) level H_2_O_2_ pretreatments; rather, we observed a significantly higher catalase activity in 6h cultures of wild-type, but not oxyR mutant, *S. marcescens* as compared to 48h cultures (stationary). It is therefore possible that rapid early growth of *Serratia* generates sufficient oxidative stress through aerobic growth to activate a stress response via oxyR. This can also explain the reduced resistance of oxyR mutants in H_2_O_2_ disk-diffusion assays [11], where the lack of an oxidative stress response makes these cells sensitive to normally sub-lethal doses of hydrogen peroxide. This oxyR-mediated protective response may well be mediated by katG. During aerobic growth of *Bradyrhizobium japonicum* [42] katG has even been shown to be the main way to remove endogenously generated H_2_O_2_, albeit without oxyR.

### Protective Mechanisms to Oxidative Stress

 The mechanism of action of ozone on bacterial cells involves oxidation of cellular components, in part through generation of reactive oxygen species such as O˙, OH˙, and hydrogen peroxide. These reactive oxygen species in turn combine with cellular lipids and proteins, causing membrane, enzyme inactivation and cell lysis [1, 27, 33]. Since hydrogen peroxide is a byproduct of ozone reactions with cells, peroxidases are reasonable means of cellular defenses against ozone [8, 33]. Indeed, defensive enzymes such as catalase have been shown to be induced by ozone exposure [9]. While our observation that exponentially growing cells have both greater ozone survival and catalase activity support a protective role of catalase, other mechanisms are certainly at work since survival of the oxyR mutant (SM-06) strain to ozone exposure was similar to that of the wild-type strain (SM-01) with a much smaller increase in catalase activity. Microbial pigments, including prodigiosin [43, 44], provide another potential protective mechanism to oxidative stress such as ozone exposure and have been shown to have antioxidant properties; although the precise mechanism of prodigiosin’s antioxidant properties is unclear, pigments such as prodigiosin act as antioxidants when they donate electrons to reactive oxygen species [45]. Our studies, however, have not found this to be the case. Other protective enzymes are also activated in response to oxidative stress. Alkyl hydroperoxide reductase (ahp) plays a critical role in removing H_2_O_2_ in *E. coli* [46] but is saturated at low (10^-5^M) levels, whereas in *Pseudomonas*, ahp is part of the oxyR regulon and is more critical in detoxifying H_2_O_2_ in biofilms [10]. Superoxide dismutase (SOD), meanwhile, converts O^2-^ into the somewhat less toxic H_2_O_2_. Genes for sodA, sodB and sodC can be found in the genome of *Serratia marcescens* [34] and at least one form of SOD has been isolated [47]. However, the role of these enzymes in *Serratia’s* oxidative stress response has been studied little and remains to be elucidated. Likewise, it remains to be determined if any of these play a role in protecting *Serratia* from ozone, studies which are now beginning in our lab.

## CONCLUSION

Although *Serratia*, when cultured at pigment-producing temperatures, showed increased survival to ozone and peroxide exposure than when cultured and assayed at higher temperatures, subsequent use of non-pigmented mutants showed no difference in ozone survival, catalase activity or hydrogen peroxide resistance. Neither does it appear that oxyR plays a role in protecting *Serratia* from ozone exposure, as oxyR mutants exhibit similar ozone survival as wild type cells. It is therefore likely that protective mechanisms other than prodigiosin or catalase, possibly regulated by temperature shift or quorum sensing mechanisms [48], contribute to *Serratia’s* resistance to both ozone and peroxide.

## Figures and Tables

**Table 1:** Strains used in this study.

Strain		Pigmentation	Reference/source
SM-R	*S. marcescens* wild type cultured at 30°C	Yes	Presque Isle Cultures
SM-W	*S. marcescens* wild type cultured at 37°C	No	Presque Isle Cultures
SM-01	*S. marcescens* wild type	Yes	Shanks Lab
SM-02	*S. marcescens*: pigA mutant	No	Shanks Lab
SM-04	*S. marcescens* Nima	Yes	Shanks Lab
SM-05	*S. marcescens* Nima: pigA mutant	No	Shanks Lab
SM-06	*S. marcescens*: oxyR mutant	Yes	Shanks Lab [11]

**Table 2:** Catalase activity of *S. marcescens* cell lysates following centrifugation to separate cell debris (pellet) from soluble components (supernatant). Activity was determined in stationary phase cells (48h cultures, N = 6) or exponential phase cells (6h cultures, N = 7) as units/ml as well as normalized to sample protein concentrations (U/mg). Protein concentrations were not determined for pelleted debris due to catalase activities indistinguishable from background.

	Stationary			Exponential
Strain	Supernatant (U/ml)	Pellet (U/ml)	Supernatant (U/mg)	Supernatant (U/mg)
SM-R	103.96±3.21	2.28±0.47	779.37±204.17	ND
SM-W	123.89±21.39	4.46±1.04	852.28±111.46	ND
SM-01	149.49±25.49	4.50±0.82	522.26±31.64	749.79±67.24 ^a,b^
SM-02	135.32±17.35	3.81±0.79	654.1±3.94	904.63±50.28 ^a,b^
SM-06	84.17±11.76	2.06±0.14	541.43±49.98	625.59±19.66

^a^: significantly different from SM during stationary phase; ^b^: significantly different from SM-06; ND: not determined
